# Correction to: #3739 Shunt portosystemic complicated by hyperammonemia and hepatic encephalopathy supported by daily dialysis in a chronic hemodialysis patient

**DOI:** 10.1093/ndt/gfad141

**Published:** 2023-07-04

This is a correction to: Nabil Hamouche and others, #3739 SHUNT PORTOSYSTEMIC COMPLICATED BY HYPERAMMONEMIA AND HEPATIC ENCEPHALOPATHY SUPPORTED BY DAILY DIALYSIS IN A CHRONIC HEMODIALYSIS PATIENT, *Nephrology Dialysis Transplantation*, Volume 38, Issue Supplement_1, June 2023, gfad063d_3739, https://doi.org/10.1093/ndt/gfad063d_3739

In the originally published version of this manuscript, there was an transposition of level figures, in **Case Report** section, 3rd paragraph, second sentence should read: “Pre-dialytic ammonia levels varied between 103 and 161 µmol/l as well as post-dialytic ammonia levels varied between 24 and 37 µmol/l, […]” instead of: “Pre-dialytic ammonia levels varied between 24 and 37 µmol/l, as well as post-dialytic ammonia levels varied between 103 and 161 µmol/l, […]”.

This error has been corrected in the article.

